# The Effects of Different Extraction Methods on Antioxidant Properties, Chemical Composition, and Thermal Behavior of Black Seed (*Nigella sativa* L.) Oil

**DOI:** 10.1155/2016/6273817

**Published:** 2016-08-25

**Authors:** Nameer Khairullah Mohammed, Mohd Yazid Abd Manap, Chin Ping Tan, Belal J. Muhialdin, Amaal M. Alhelli, Anis Shobirin Meor Hussin

**Affiliations:** ^1^Faculty of Food Science and Technology, Universiti Putra Malaysia (UPM), 43400 Serdang, Selangor, Malaysia; ^2^Department of Food Science and Biotechnology, College of Agriculture, University of Tikrit, Tikrit, Iraq; ^3^Halal Products Research Institute, Universiti Putra Malaysia (UPM), 43400 Serdang, Malaysia

## Abstract

The* Nigella sativa* L. popularly referred to as black seeds are widely used as a form of traditional nutrition and medicine.* N*.* sativa* seeds were used for the extraction of their oil by way of supercritical fluid extraction (SFE) and cold press (CP) to determine the physicochemical properties, antioxidant activity, and thermal behavior. The GC-MS results showed the primary constituents in the* Nigella sativa* oil (NSO) were Caryophyllene (17.47%) followed by thymoquinone (TQ) (11.80%), 1,4-Cyclohexadiene (7.17%), longifolene (3.5%), and carvacrol (1.82%). The concentration of TQ was found to be 6.63 mg/mL for oil extracted using SFE and 1.56 mg/mL for oil extracted by CP method. The antioxidant activity measured by DPPH and the IC_50_ was 1.58 mg/mL and 2.30 mg/mL for SFE oil and cold pressed oil, respectively. The ferric reducing/antioxidant power (FRAP) activity for SFE oil and CP oil was 538.67 mmol/100 mL and 329.00 mmol/100 mL, respectively. The total phenolic content (TPC) of SFE oil was 160.51 mg/100 mL and 94.40 mg/100 mL for CP oil presented as gallic acid equivalents (GAE). This research showed that a high level of natural antioxidants could be derived from NSO extracted by SFE.

## 1. Introduction

Black seed (*Nigella sativa* L.), traditionally used to treat fever, headache, anxiety, diarrhea, asthma, and stroke, is known to be highly anti-inflammatory [1, 2].* N*.* sativa* seed is rich in phenolic compounds used as an antioxidant agent [1] and in essential fatty acids besides bioactive compounds such as sterols and tocols [3]. Moreover, the yellowish oil contains proteins, amino acids, reducing sugars, mucilage, alkaloids, organic acids, tannins, resins, toxic glucoside, metarbin, bitter principles, glycosidal saponins, crude fiber, minerals, and vitamins [4]. Among the various oil seeds,* N*.* sativa* oil (NSO) is particularly interesting as it may be utilized in preparations that contain phytochemicals with strong antioxidant properties and health benefits [5, 6]. Thymoquinone (TQ) is an active compound in the crude extracts of NSO, which possesses antioxidant/anti-inflammatory efficacy in models of* in vitro* and* in vivo* investigations as well as asthma, diabetes, encephalomyelitis, neurodegeneration, and carcinogenesis [5, 7].

Methods of extraction of seed oils are an effective factor in the properties of oils. Solvent extraction, for example, is deficient in selectivity and needs extreme heat, which could cause the degradation of the desired components [8]. The cold press extraction is the conventional method for oil extraction. It involves no heat and/or chemicals and this is preferred by consumers concerned about natural and safe food [9]. However, this method affords low yields [10], and the residual meal contains 10–12% oil content, which can eventually limit its uses in industries processing food [11]. Supercritical fluid extraction (SFE) is currently a technique among others used to extract plant oils and offers some favorable features over the traditional techniques that have been used in the oil industry [12]. In general, SFE has been the recommended method used to extract antioxidant compounds from NSO and exhibits a higher concentration of thymoquinone [13].

This study aims to compare the properties of NSO extracted using SFE and cold press with regard to the antioxidant activity, chemical composition, TQ concentration, and the thermal behavior of the oil.

## 2. Experimental

### 2.1. Materials and Methods

*N*.* sativa* seeds were sourced from the Serdang spice market in West Malaysia. Analytical-grade solvents, thymoquinone (99.9% purity) and 1,1-diphenyl-picryl-hydrazyl, came from Sigma-Aldrich (St. Louis, MO, USA).

### 2.2. Chemical Properties of* Nigella sativa* L. Seeds

Moisture (930.04), ash (930.05), and protein (978.04) contents were established based on [17]. Fat determination was done using Soxhlet extractor and hexane as the solvent. In brief, 15 g of seed were ground and fat was obtained after 8 h of extraction and the results were presented as the amount in percent of lipids in the dry matter of seed powder, while estimation of the carbohydrate content achieved was expressed in terms of the variance of the mean values, that is,

(1) $$\begin{array}{c} \left[\;100-\left(\;\text{protein}+\text{lipids}+\text{ash}+\text{moisture}\;\right)\;\right]. \end{array}$$

### 2.3. Oil Extraction

#### 2.3.1. Cold Pressing

The* N*.* sativa* L. seeds were pressed at room temperature (25°C) by mechanical pressing without any heating treatment. Crushed seeds were stored for one night at room temperature to separate oil phase from fibers, and then oil was filtered using Watman #4 filter paper and a glass funnel.

#### 2.3.2. Supercritical Fluid Extraction

Extracting essential oil from* N*.* sativa* L. seed was performed using the SFE instrument (FeyeCon Development B. V., Netherland) at the supercritical fluid centre, Faculty of Food Science and Technology, UPM, as described by [18], with some modification. The dried seeds were completely crushed for 3-4 min in a stainless steel grinder (Waring Commercial, Torrington, CT, USA) and kept in a 50-liter extractor container of the same material and tightly sealed. Supercritical fluid extractions were conducted at pressures of 600 bar and temperatures of 40°C for the duration of 1 h, and liquid CO_2_ was injected at approximately 150 L/hour and controlled by an automatic back pressure regulator.

### 2.4. Physicochemical Properties of* Nigella sativa* Oil

Peroxide value (PV), free fatty acids content (FFA), saponification value (SV), and iodine value (IV) were established based on [17]. The refractive index (RI) was calculated with Abbe Refractometer at 20°C. The viscosity of NSO was extracted using SFE and cold press was recorded at 25°C with a Haake Rheometer (model Stress 600, Thermo Electron Corporation, Karlsruhe, Germany). The rheometer is, in essence, a dynamically managed stress instrument with a standard sensor system and a plate (PP35Ti). Data received were subjected to analysis with Haake RheoWin 3 Data Manager software. The steel cone-plate (C40/4) measuring system was utilized to measure how viscous the samples were at a shear rate of 0 to 100 s^−1^. All measurements were carried out in triplicate.

### 2.5. GC-MS Test

GC-MS analyses were conducted in a gas chromatograph Trace GC Ultra gas chromatograph attached to a TSQ Quantum XLS triple quadruple mass spectrometer, both from Thermo Scientific (Waltham, MA, USA). All the analyses were performed with a fused silica DB-5-MS column (30 m × 0.25 mm × 0.25 *μ*m), which was employed for all the analyses. The temperature of the oven was raised to 220°C from 70°C at 4°C/min and maintained isothermally for 15 min. The temperatures of the injector and detector temperatures were maintained at 220°C and 240°C, respectively, with the preparation of 10% of samples in acetonitrile. The split mode ratio of 1 : 15 was applied for the injection of a 0.5 *μ*L sample. Carrier gas used was helium at 1 mL/min flow rate. Other parameters were kept the same in relation to GC analyses. EI at 70 eV provided the mass spectra with mass scanning done from 40 to 400 amu. Percentage of each component was calculated based on GC peak areas.

### 2.6. HPLC Quantification of Thymoquinone

Thymoquinone evaluation of NSO was conducted along the lines of the approach taken by [19] with minor modification. The oils obtained from SFE and cold press extraction methods were passed through a C18 column preeluted with methanol. 20 *µ*L of NSO followed by 800 *µ*L (400 *µ*L × 2) methanol was added to give an eluate free from greasy and fatty materials. The analysis was carried out on the oils from SFE and cold press extraction methods for TQ with the use of Agilent 1200 HPLC system (Agilent Technologies, Palo Alto, USA) with a diode array detector (HPLC-DAD). A Prevail C18 column (250 × 4.6 mm ID, with 5 *μ*m particle size, Agilent Technologies, USA) was employed with a mobile phase of water : methanol : 2-propanol (50 : 45 : 5% v/v) which was filtered using a 0.45 mm Millipore filter and 20 *μ*L was the volume of the injection. Analysis of thymoquinone was detected at 254 nm at room temperature. A 1.5 mL/min flow rate was used and identification was confirmed through a comparison of the time, the standard compound was retained with that of oil sample and the quantity calculations were achieved by constructing the standard linear calibration curves.

### 2.7. 1, 1-Diphenyl-2-picrylhydrazyl (DPPH) Radical Scavenging Activity Test

This assay was performed according to [20] with some changes made to establish the radical scavenging activity of NSO. The DPPH solution was prepared fresh as and when it was needed by diluting 2.5 mg DPPH in 100 mL methanol. Then, the mixture of 0.25 mL of NSO and 1.75 mL methanolic DPPH was prepared in a 96-wall plate. Following 30 min of incubation in the dark and at ambient temperature (25°C), the absorbance was measured at 515 nm, the wave length of highest absorbance of DPPH was noted with an ELISA reader (labomed, model UVD-2950, USA). A blank experiment or control was done applying similar steps to a solution minus the test material. The absorbance was recorded as *A*. The free radical scavenging activity of all solutions was then calculated as percentage of inhibition on the basis of the following equation:

(2) $$\begin{array}{c} \%\;\text{inhibition}=\frac{\left(A-B\right)}{A}*100, \end{array}$$

where *A* = absorbance of control and *B* = absorbance of the sample.

Antioxidant activities of test compounds were expressed as IC_50_, which is represented by the amount of antioxidant compounds that caused 50% scavenging of DPPH radicals for the duration of the defined time frame.

### 2.8. FRAP Assay

The ability of the NSO antioxidant of the ferric reducing power was assessed with (FRAP) assay as indicated by [21]. Briefly, FRAP reagent was freshly prepared, and 20 mM FeC_l3_ and 10 mM TPTZ solutions were mixed in 40 mM HCl and 300 mM acetate buffer (pH 3.6) in ratios of 1 : 1 : 10 (v/v/v). Then, 10 *μ*L of sample (dissolved 100 *μ*g in 1 mL methanol) was distributed into a 96-well plate, and then 200 *μ*L of FRAP reagent was placed into the same well containing the sample. The solution was mixed and incubated for 30 min at room temperature. The absorbance was recorded at 593 nm using ELISA reader (Power Wave ×340, BioTek Instruments, Inc., Winooski, VT, USA). Results of FRAP were expressed in mM of ferrous equivalents, Fe (II) per g three times and readings were taken in triplicate.

### 2.9. Total Phenolic Content

The entire amount of phenolic compounds (TPC) of NSO was established with the use of Folin-Ciocalteu reagent (FCR) according to [22] with certain changes. There was the addition of 0.5 mL sample to 0.5 mL of Folin-Ciocalteu reagent, and 5 minutes later, 10 mL of 7% of aqueous Na_2_CO_3_ sodium carbonate solution was added and mixed to react then kept for 1 hour in total darkness at room temperature. The absorbance was read at 725 nm employing ELISA reader (Power Wave ×340, BioTek Instruments, Inc., Winooski, VT, USA). A standard curve of gallic acid was separately made from versus concentration (0-1 mg/mL). The outcomes were presented in gallic acid equivalents (GAE) mg/kg dry weight. All experiments were conducted three times and reading was obtained in triplicate.

### 2.10. FTIR Spectroscopy

Fourier-transform infrared (FTIR) spectroscopy of NSO extracted by either SFE or cold press machine was carried out on a Spectrum 100 FTIR spectrometer from Perkin-Elmer Corporation, USA. FTIR for the samples was recorded over the range of 400–4000 cm^−1^ employing a sample of approximately one percent in 200 mg of spectroscopic-grade potassium bromide (KBr) with 10 tons of pressure.

### 2.11. Thermal Behavior DSC

Thermal properties of NSO extracted by SFE and cold press were examined by differential scanning calorimeter using DSC 820e from Mettler Toledo (Schwerzenbach, Switzerland). Weighing of the oil samples was done (8-9 mg) straight into a DSC-pan (SFI-Aluminium, TA Instrument T11024). An empty aluminium pan, hermetically sealed, was the reference. The samples were heated from 30°C with a speed of 5°C/min to 90°C. The same process was repeated and recording of the DSC thermographs was done during the second melting. The DSC manufacturer's software (STARe Speciality Library) was employed to analyse the data of the heat flow and the exact heat of the oil samples was calculated. Results were derived by averaging triplicate samples.

### 2.12. Thermogravimetric Analysis (TGA)

Thermogravimetric method determines the thermal properties of oils as a function of temperature. Examination of the thermally induced degradation of the oils was conducted in a thermogravimetric analysis (TGA) Perkin-Elmer Thermogravimetry Analyzer Pyris 2. The analysis was carried out on approximately 25 mg of samples at the temperature range of 25–1000°C at a constant heating rate of 25°C/min in an atmosphere of nitrogen.

### 2.13. Statistical Analysis

The considerable variance of the mean values was confirmed using Tukey's test. A probability of *p* > 0.05 was deemed significant. The statistical software, MINITAB 16 (Minitab Inc., State College, PA, USA) was employed for all the analyses.

## 3. Results

### 3.1. Physicochemical Properties of* Nigella sativa* L. Seed

The approximated structure of the* N*.* sativa* seed powder and related literature references are presented in Table 1. The analysis of the* N*.* sativa* L. seed demonstrated its contents to be 32.37% lipids, 6.78% moisture, 19.23% protein, 6.94% ash, and 35.08% carbohydrate. FFA values of NSO extracted by SFE and CP methods were significantly different with values of 6.15 and 5.98% (as oleic acid), respectively (Table 2). The iodine values were 115 g of I2/100 g of oil for SFE and 104 g of I2/100 g of oil for CP oil (Table 2). The SFE and CP extracted oils showed the saponification values of 243.52 (mg of KOH/g of oil) and 238.26 (mg of KOH/g of oil), respectively (Table 2). Table 2 presents the refractive index (RI) of NSO extracted by SFE and cold press 1.47813 and 1.47719, respectively. Peroxide values (PV) of oils obtained by using SFE and cold pressing extraction were 3.4 and 4.1 meq O_2_/kg oil, respectively.

### 3.2. GC-MS Studies

**Figure 1:**
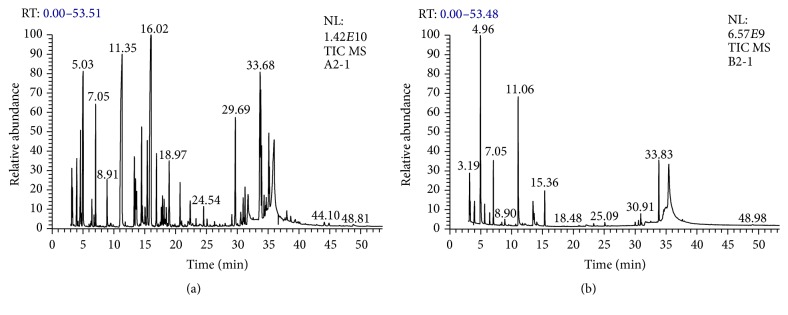
GC-MS chromatography analysis of* Nigella sativa* oil extracted by (a) 474 supercritical fluid and (b) cold press.

The results showed significant differences between the SFE and the cold press oils based on the peak area (Table 3). Twenty compounds were identified in the SFE oil, while the cold press oil had 19 compounds. The major components of the SFE oil were Caryophyllene (17.47%) followed by thymoquinone (16.80%), 1,4-Cyclohexadiene (7.17%), longifolene (3.5), and carvacrol (1.82%) (Figure 1(a)). The major components for cold press oil were 1,3,8-p-Menthatriene (23.82%) followed by thymoquinone (16.21%), 1,4-Cyclohexadiene (7.17%), longifolene (4.49), and carvacrol (3.90%) (Figure 1(b)).

**Figure 2:**
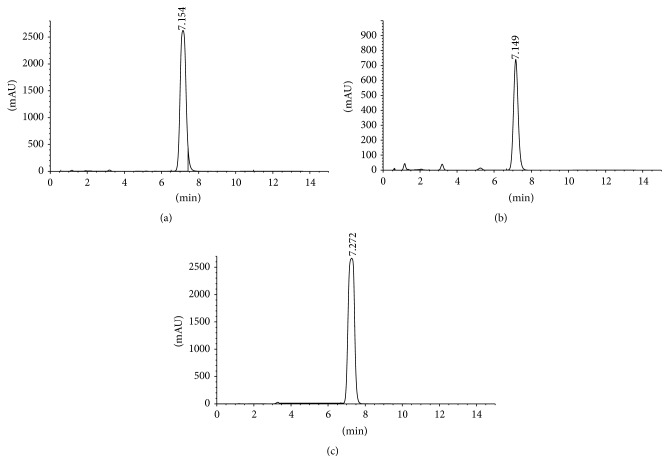
Thymoquinone concentration of* Nigella sativa* oil as determined by HPLC: (a) 490 supercritical fluid extraction, (b) cold press extraction, and (c) thymoquinone standard.

The HPLC analysis showed that the two oil samples (SFE and cold pressed) contained thymoquinone TQ (Figures 2(a) and 2(b)). The highest amount of TQ was observed in the SFE oil and was 6.63 mg/mL of oil, while the concentration of TQ was 1.56 mg/mL oil for the cold press sample (Table 4). The methods of extraction showed important differences (*p* > 0.05) for the SFE with higher thymoquinone quantity.

### 3.3. Antioxidant Activity of* Nigella sativa* L. Oil

The antiradical activity of NSO to scavenge DPPH demonstrated high levels with SFE oil IC_50_ being 1.5873 mg/mL while the CP oil IC_50_ was 2.3086 mg/mL (Table 5). The reducing ability of NSO extracted by two methods, SFE and cold press, was in the range of 329.00–538.67 mol/100 mL oil (Table 5). The concentration of overall phenolic compounds (TPC) in NSO extracted by SFE and CP was assessed using Folin-Ciocalteu method presented as gallic acid equivalents as can be seen in Table 5. Results in this table show a considerable difference (*p* < 0.05) between the overall phenolic content of the SFE oil with a value of 160.51 mg GAE/100 mL and CP oil with a value of 94.40 mg GAE/100 mL (Table 5).

### 3.4. FTIR Spectra of* Nigella sativa* L. Oil

**Figure 3:**
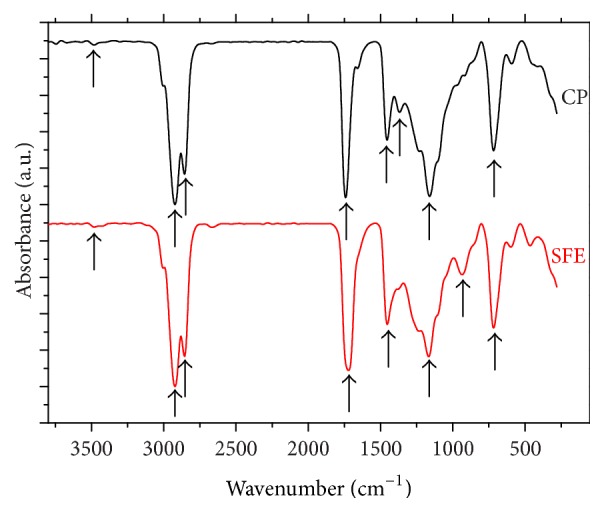
FTIR spectra of* Nigella sativa* L. oil scanned at 4,000–650 cm^−1^: supercritical fluid (SFE) and cold press (CP).

Figure 3 reveals FTIR spectrum of SFE and CP oils at mid infrared region (4,000–650 cm^−1^). In this study, the spectrum of both oils showed very close features of absorption bands identical to the most common triglyceride molecules with certain fatty acids (Table 6).

### 3.5. Thermal Behavior of* Nigella sativa* L. Oil

**Figure 4:**
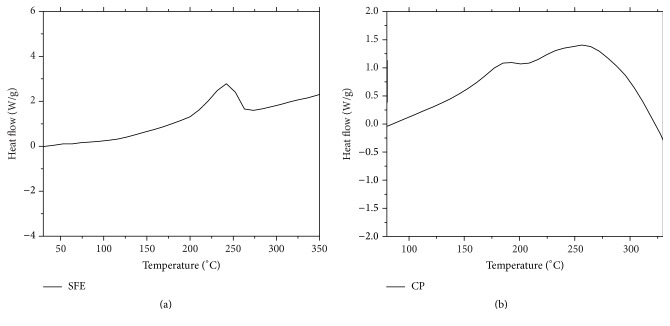
Differential scanning calorimetry (DSC) thermal behavior of* Nigella sativa *oil: 529 (a) supercritical fluid and (b) cold press.

These curves of the SFE and CP oils showed a plain thermogram with one peak possessing features such as onset temperature at 203.66°C and 243.03°C, melting enthalpy is 30.48 J/g and 34.50 J/g, and melting peak 241.04°C and 264.35°C, respectively (Figures 4(a) and 4(b)).

**Figure 5:**
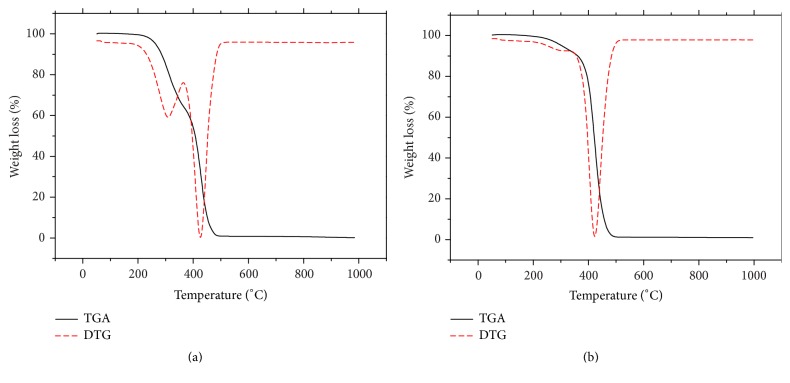
TGA and DTG curves of weight loss of* Nigella sativa *oil: (a) supercritical fluid 540 and (b) cold press.

The weight loss occurred in SFE oil in the percentage of 35.1 and 63.4% at 208 and 365°C, respectively (Figures 5(a) and 5(b)). TGA and DTG curves for the SFE oil showed the degradation in two steps, first point at 308.5°C and second degradation at 365.5°C, while for the CP oil had weight loss of 6.3 and 91.4% at 212.4 and 33.3°C, respectively. In addition, degradation points have been observed in CP oil at 288.3°C and 423.77°C, respectively.

## 4. Discussion

The analysis of the* N*.* sativa* L. seed demonstrated that its contents of lipids and moisture were in the range of those found in the literature, while protein was slightly lower and ash with carbohydrate (by difference) were higher compared to those revealed in the literature [13–16]. The chemical properties of oils are considered the most significant feature to determine the quality of oil samples.

In this work, chemically, the properties of* N*.* sativa* L. oil extracted using SFE and cold press were studied. Free fatty acid and peroxide value are the commonly used indicators to monitor the quality of seed oils. In the current study, the low FFA value demonstrated that oil extracted with the SFE method had higher stability than that obtained by CP extraction. This finding is in agreement with the oil obtained through SFE method (5.83%) and Soxhlet method (6.40%) in [13] and much less compared to cold pressed oil (11%) [14]. In another study, the iodine value was higher than that of this study's results according to [23] for SFE oil (127 g of I_2_/100 g of oil) and (121 g of I_2_/100 g of oil) for Soxhlet oil. The iodine index shows that NSO is an extremely unsaturated oil, suggesting that it possesses elevated levels of oleic and linoleic acids, with the lower iodine value contributing to the high stability of the oil [14]. The saponification values (SV) of NSO extracted by SFE and CP methods were high compared to other seed oils suggesting the presence of high triacylglycerols content. The saponification values in this study were comparable to the values of Tunisian 211 (mg of KOH/g of oil) and Iranian 218 (mg of KOH/g of oil) extract from CP extraction with hexane reported by [14]. The result of refractive index (RI) was similar to those reported previously, while the peroxide values (PV) of oils were lower than cold press 13.5 meq O_2_/kg oil reported by [15]. There could be a relation between these differences and the dissimilarities of cultivated regions, maturity stage, and storage circumstances. According to the data reported about the nutritional value of* N*.* sativa* seed, it is noted that besides the high oil content it is also a rich source of many minerals and bioactive compounds. Compounds were recognized through comparison of their retention indices and mass obtained in GC-MS chromatogram (Figures 1(a) and 1(b)) with those of NIST02 library data stored in the computer library and Adams libraries spectra [24, 25] and enumerated along with molecular weight, retention time, and peak area. Burits and Bucar's [1] method for the extraction of essential oil used light petroleum followed by steam distilling of the extract. They indicated that their essential oil possessed thymoquinone as the main component, while in this study Caryophyllene is the major component of the SFE oil and 1,3,8-p-Menthatriene for cold press oil.

The high content of thymoquinone could be because of the greater selectivity of the SFE method compared to cold press. Generally, extractions using cold press result in partial oil recovery [26]. In fact, the cold press gives low yields, and usually the extraction process is time consuming [10] which may affect the concentration of the target compounds from NSO quantity. The results for thymoquinone quantity were similar to those of Solati et al. [13] who reported that the NSO extracted by SFE had a high content of TQ with 4.09 mg/mL. Furthermore, Ismail et al. [18] reported that NSO extracted with SFE was rich in thymoquinone with the percentage of about four times higher compared to the oil extracted by conventional solvent extraction method. TQ is a phytochemical compound of NSO, which is interesting for study as it contributes to the oil's general stability and potential health benefits [27]. It has also been shown to be the reason for the biological antioxidant activity of NSO and also for the majority of the beneficial health effects related to the seeds and oils [13].

The antiradical activity of NSO to scavenge DPPH was determined based on their IC_50_ values, described as the quantum of the antioxidant needed to inhibit 50% of DPPH existing in the test material. Both the SFE and CP oils had high ability to decrease the stable radical DPPH. The earlier studies by [1, 13] reported higher IC_50_ (lowest antioxidant activity) compared to this study's IC_50_ of 2.26 mg/mL. These data confirm that SFE is a more appropriate method of extraction for NSO. The SFE oil exhibited a high antioxidant power corresponding to that of CP oil. The ferric reducing ability (FRAP assay) is very often employed to evaluate the antioxidant component in dietary polyphenols [28]. Antioxidant activity is known to have a linear proportion of the phenolic contents. Oktay et al. [29] mentioned the likelihood of total phenolic contents and antioxidant activity being very positively related between, a trend that seems to be prevalent in several species of plants. Sielicka and Samotyja [30] evaluated several oils for ferric reducing activity, and among the tested oils, the highest activity was observed for NSO, which ranged between 678 and 2102 *μ*mol/100 g oil. Compared to this study, the results are significantly higher for both CP oil and SFE oil. The SFE oil exhibited the highest FRAP activity which indicated the advantages of the SFE method.

Polyphenolic compounds are acknowledged to be antioxidant active and there is a likelihood that these compounds are the cause of abstracts activity [31]. In other studies, total phenolic contents of NSO extracted by solvent from Tunisian and Iranian* Nigella sativa* seeds were 245 and 309 mg/kg expressed as gallic acid, respectively [14]. Viuda-Martos et al. [32] determined the TPC in NSO and observed the high content of phenolic compounds (726.67 mg/L) in the commercial oil, and these results mirror those of CP oil in this study, while these findings were much less than the oil extracted by SFE. This indicates that using SFE for the extraction of the oil is preferred as it will yield a higher concentration of phenolic compounds.

Differential scanning calorimetry DSC offers evidence of the additional specific heat across a broad temperature range [33]. Any endothermic or exothermic event is noted as a peak in the chart, and its area is in proportion to the enthalpy achieved or lost, respectively. Figures 4(a) and 4(b) show the DSC melting curves for NSO extracted by two different extraction methods. The melting curves of these seed oils show more endothermic peaks and shoulders. In general, both NSO samples showed similar DSC melting point and profiles higher than 200°C temperature regions. This result concurs with published data [14]. Thermogravimetric analysis (TGA) and derivative thermograms analysis (DTG) were conducted to determine the thermal stability of two samples for NSO extracted by SFE and cold press. Thermogravimetric analysis (TGA), also referred to as thermogravimetry (TG), is a technique where measuring the thermal behavior of mass as a function of heating is recorded. The TGA and the DTG curves revealed an inverse linear correlation between the weight and the temperature as the NSO lost weight with an increase in the temperature. From TGA profile of both oils, there is no weight loss observed before 200°C. TGA and DTG curves' behavior remained similar for the CP oil which showed similar thermal behavior with SFE oil. This might be due to moisture evaporation and the presence of other volatiles in oil samples. The onset temperature and peak value of the SFE oil compared to CP oil are also similar. Thus, it is clear that the thermal stability of the* N*.* sativa* seed oils was not significantly affected by the type of extraction method.

## 5. Conclusion

In conclusion, the NSO extracted by using supercritical fluid had a high concentration of thymoquinone and total phenolic compounds. Moreover, the antioxidant activities were high with low IC_50_ value as established by DPPH and FRAP assays. The oil extracted by CP and SFE had no significant differences in their thermal profiles with no weight loss observed before 200°C and both oils have the same functional groups. The GC-MS showed significant differences in the two oil profiles which are directly affected by the extraction method employed. The SFE method can be considered as the optimum process for the extraction of NSO since the method enhances the quality of the yield in comparison with CP.

## Figures and Tables

**Table 1:** Chemical properties of the selected *Nigella sativa *seed in comparison with previous studies.

Total fat	Moisture	Ash	Protein	Carbohydrate	Studies
32.26 ± 0.09	6.67 ± 0.20	6.82 ± 0.10	19.19 ± 0.20	35.04 ± 0.30	Current study
31.72 ± 0.42	4.99 ± 0.29	5.29 ± 0.41	23.07 ± 1.05	34.91 ± 1.22	Solati et al., 2014 [13]
40.35 ± 0.16	—	4.41 ± 0.01	22.6 ± 0.24	32.7 ± 0.41	Cheikh-Rouhou et al., 2007 [14]
34.8 ± 1.90	7.0 ± 0.50	3.7 ± 0.70	20.8 ± 1.10	33.7 ± 0.50	Atta, 2003 [15]
37.33 ± 0.15	5.40 ± 0.13	6.72 ± 0.02	20.02 ± 0.27	30.53	Khoddami et al., 2011 [16]

**Table 2:** Oil physiochemical properties obtained using two extraction methods.

Physiochemical properties	Supercritical fluid extraction	Cold press extraction
FFA (as oleic %)	5.98 ± 0.00^b^	6.15 ± 0.00^a^
SV (mg of KOH/g of oil)	243.52 ± 0.3^a^	238.26 ± 0.67^b^
IV (g of I_2_/100 g of oil)	115.1 ± 0.24^a^	104.37 ± 0.43^b^
PV (meq O_2_/kg oil)	3.4 ± 0.05^a^	4.1 ± 0.15^b^
Refractive index at (25°C)	1.47 ± 0.00^a^	1.47 ± 0.00^a^
Viscosity (mPa s)	6.26 ± 0.07^a^	6.38 ± 0.08^a^

Means of triplicate measurements in the same row ± standard deviation with different letters are significantly different (*p* < 0.05).

**Table 3:** GC-MS identification of chemical composition for *Nigella sativa *oil extracted by supercritical fluid and cold press.

	Compound name	Supercritical fluid			Cold press		
		MW	RT	Peak area (%)	MW	RT	Peak area (%)
1	*α*-Pinene	—	—	—	136	3.19	7.10
2	3-Carene	136	3.29	2.60	136	3.29	2.21
3	Bicyclo[2.2.1]heptane	136	4.00	2.05	136	4.00	3.67
4	1,3,8-p-Menthatriene	—	—	—	134	4.96	23.82
5	ç-Terpinene	—	—	—	136	5.64	2.82
6	1,4-Cyclohexadiene	134	5.01	7.17	—	—	—
7	cis-4-Methoxy thujane	168	6.45	3.09	168	7.05	7.30
8	Cyclohexen	154	8.90	3.82	—	—	—
9	Limonene	—	—	—	236	8.41	0.41
10	Thymoquinone	164	11.32	16.80	164	11.06	16.21
11	Carvacrol	150	13.51	1.82	150	13.46	3.90
12	Longifolene	204	13.70	3.50	204	15.37	4.49
13	Ylangene	204	14.50	2.99	204	13.67	0.82
14	3-Allyl-6-methoxyphenol	164	14.61	0.40	—	—	—
15	1-Heptatriacotanol	—	—	—	536	20.91	0.14
16	2,3-Dihydrofarnesyl acetate	—	—	—	266	30.91	1.54
17	Caryophyllene	204	16.03	17.45	—	—	—
18	Naphthalene	204	17.88	2.94	—	—	—
19	*α*-Bisabolene	204	18.50	0.50	—	—	—
20	(−)-Spathulenol	220	22.37	0.79	—	—	—
21	Methyl tetradecanoate	242	24.54	0.54	—	—	—
22	Hexadecanoic acid	254	29.69	4.99	254	31.65	0.77
23	Ascorbic acid	652	31.75	1.12	652	34.95	2.15
24	9,12-Octadecadienoic acid (Z,Z)	294	33.70	14.05	294	35.45	7.85
25	4,8-Decadienal, 5,9-dimethyl	—	—	—	180	33.84	8.12
26	Methyl stearate	298	34.34	0.61			
27	Butyl 9,12-octadecadienoate	336	35.11	2.29	336	11.77	0.21
28	Cyclopropanebutanoic acid	—	—	—	374	37.66	0.25

	Total			89.54			93.53

MW: molecular weight.

RT: retention time.

**Table 4:** Thymoquinone concentration of *Nigella sativa *oil extracted by supercritical fluid and cold press as determined by HPLC.

Extraction methods	Thymoquinone
SFE oil	6.37 ± 0.00^a^ mg/mL
CP oil	1.78 ± 0.00^b^ mg/mL

± Standard deviation of three replications.

^ab^Different letters in the same column represent significant (*p* < 0.05) differences.

**Table 5:** Antioxidant activities of *Nigella sativa *oil determined by DPPH radical scavenging and FRAP ferrous reducing, with TPC total phenolic content.

Types of oil	DPPH IC_50_ (mg/mL)	FRAP (mmol/100 mL)	TPC (mg/100 mL GAE)
Supercritical fluid oil	1.58 ± 0.07^b^	538.67 ± 12.58^a^	160.51 ± 11.43^a^
Cold press oil	2.30 ± 0.02^a^	329.00 ± 54.78^b^	94.40 ± 1.02^b^

± Standard deviation of three replications.

^ab^Different letters in the same column represent significant (*p* < 0.05) differences.

**Table 6:** The main peaks in the FTIR spectrum of *Nigella sativa* oils extracted by supercritical fluid of and cold press with their assignment.

Oil types	Peak (cm^−1^)	Functional group
SFE-CP	3482	Primary amines (-NH_2_ groups)
SFE-CP	2922, 2856	C-H stretching vibration (aliphatic) (CH_3_)
SFE-CP	1721	C=O stretching vibration (ester)
SFE-CP	1456, 1367.74	C-H bending vibration (aliphatic) (CH_2_)
SFE-CP	1166.48	C-O stretching vibration (ester)
SFE-CP	935.42, 717.35	trans-CH=CH-
